# The Modum-ED Trial Protocol: Comparing Compassion-Focused Therapy and Cognitive-Behavioral Therapy in Treatment of Eating Disorders With and Without Childhood Trauma: Protocol of a Randomized Trial

**DOI:** 10.3389/fpsyg.2019.01638

**Published:** 2019-07-18

**Authors:** KariAnne R. Vrabel, Bruce Wampold, Daniel S. Quintana, Ken Goss, Glenn Waller, Asle Hoffart

**Affiliations:** ^1^Research Institute of Modum Bad, Vikersund, Norway; ^2^Department of Counseling Psychology, University of Wisconsin, Madison, WI, United States; ^3^NORMENT, Division of Mental Health and Addiction, KG Jebsen Centre for Psychosis Research, Oslo University Hospital, Oslo, Norway; ^4^Institute of Clinical Medicine, University of Oslo, Oslo, Norway; ^5^Coventry Eating Disorder Service, Coventry, United Kingdom; ^6^Department of Psychology, University of Sheffield, Sheffield, United Kingdom

**Keywords:** eating disorders, RCT, CBT, CFT, outcome, process, childhood trauma

## Abstract

**Background:** The combination of eating disorder (ED) and the experience of childhood trauma leads to significant impairment and suffering. To improve treatment, it is critically important to study treatment effects, and the mechanism of these effects. The overall aim of the current project is to; (1) build knowledge on how to best treat patients with ED with and without childhood trauma, (2) develop our understanding about how change happens for these patients. We will do this by comparing two treatment models in an inpatient setting; Compassion-Focused Therapy (CFT) and cognitive-behavioral therapy (CBT) for ED. This paper describes the development, design and implementation of the trial.

**Methods and Design:** Patients included in this randomized controlled trial will satisfy DSM-5 criteria for ED and approximately half of the patients will in addition have a history of childhood trauma. A total of 144 patients who have received either CFT or CBT are followed up 1 year after completion of the treatment. The study will collect a rich dataset of outcome measures at four time points, and process and sub-outcome measures at 13 time points. All patients will be assessed with the same clinical instruments based on current state-of-the-art methods. The primary outcome will be change in the severity of ED features as measured by the global ED examination score, and having a global ED examination score less than one standard deviation above the community mean, while secondary outcomes will relate to treatment effects on trauma symptoms, general symptoms, and quality of life.

**Discussion:** This trial will make an important contribution to the need for evidence of effective treatment for patients with ED with or without childhood trauma.

**Ethics and Dissemination:** The project is approved by the South-Eastern Regional Committee for Medical and Health Research Ethics of Norway (REC;2014/836).

**Clinical Trial Registration:** ClinicalTrials.gov, http://www.Clinicaltrials.gov/ct2/show/NCT02649114.

## Introduction

Eating disorders are severe mental disorders (Hudson et al., 2007; Jenkins et al., 2011; Mitchison et al., 2012; Stice et al., 2013). In the fifth edition of the diagnostic and statistical manual of mental disorders (DSM-5) (American Psychiatric Association [APA], 2013) there are five EDs: (a) AN, (b) BN, (c) BED and two categories derived from the previous diagnosis ED not otherwise specified (d) other specified feeding or eating disorder (OSFED), and (e) unspecified feeding or eating disorder (USFED) (Attia et al., 2013).

The treatment research on EDs has focused on BN and, more recently, BED. Studies of high quality treatment for OSFED and AN are limited. This is a challenge, as OSFED has been the highest prevalent ED diagnosis in routine clinical practice (Fairburn and Bohn, 2005; Machado et al., 2007).

Clinical guidelines recommend cognitive behavioral therapy (CBT) as the psychological treatment of first choice, specifically for BN, and BED (National Collaborating Centre for Mental Health, 2004; American Psychiatric Association [APA], 2006). There are few specific therapy recommendations for AN due to the scarcity of conclusive outcome data for these patients. Clinical aims in CBT include decreasing the core beliefs about low self-worth and compensatory beliefs about the need to control food intake, body weight, and shape. Use of stimulus-control procedures to reduce the frequency of disordered eating, is central (Waller et al., 2007). Thus, the aims of CBT are behavioral change and changes in mood and cognitions. Studies, systematic reviews, and meta-analyses show promising effects of CBT for BED, and intermediate effects for BN, but the methodological quality of studies is low to moderate (Hay, 2013; Spielmans et al., 2013).

However, up to 50% of patients with ED do not recover after CBT (Wilson et al., 2007). The highest probability of poor treatment outcomes in patients with ED has been observed in those who experienced childhood sexual abuse and exposure to violent acts at an early age (Mahon et al., 2001; Rodriguez et al., 2005). A study showed that the risk of dropout or relapse rates was, respectively, 10 and 3 times greater among patients with ED who reported previous traumatic events compared with those patients without a history of trauma (Rodriguez et al., 2005). Our own study revealed that childhood sexual abuse and avoidant personality disorder predicted non-response course for patients with longstanding ED who received CBT (Vrabel et al., 2010). Considering the poor outcome and high dropout and relapse rates for these patients, there is a need to develop treatments tailored for patients with ED with trauma histories. To date, however, no treatment approaches for this population have been empirically tested.

When individuals endure early experiences of traumatic experiences they can become more prone toward self-criticism and to develop shame, a hurtful emotional condition characterized by perceiving oneself as a failure (Gilbert, 1998). They may try to regulate their feelings of inadequacy with strategies which ease their pain for a limited time. Accordingly, ED symptoms such as restriction, binging, and compensating behavior such as purging and excessive exercising, could be viewed as efforts to protect themselves and to control underlying feelings of shame (Goss and Gilbert, 2002). ED symptoms are effective at reducing shame in the short-term, but ultimately intensify these feelings in the long-term (Goss and Allan, 2009). Studies have shown that shame and self-criticism have been shown to be a major vulnerability and maintaining factor both for ED and posttraumatic stress disorder (PTSD) (Lee et al., 2001; Irons and Gilbert, 2005) and decreased self-criticism and increased self-compassion has been found to be particularly beneficial in reducing psychological distress and improving well-being (Lutz et al., 2004; Gilbert, 2007; Hoffart et al., 2015). CFT is a transdiagnostic psychotherapeutic approach that seeks to help shame-prone highly self-critical individuals to improve an attitude of inner-kindness and self-compassion (Gilbert, 2005, 2010). Indeed, this treatment should therefore be particularly suitable for a population with ED and childhood trauma.

There is some evidence that indicate that CFT could be beneficial for patients with ED, however, only three studies to our knowledge have investigated the treatment in ED patients. The first study, found that a 3-week self-compassion intervention reduced binge eating and weight and shape concerns among individuals with BED (Kelly and Carter, 2015). In the second study, Gale et al. (2014) examined the effectiveness of a CBT group outpatient treatment where CFT were integrated in the treatment, for individuals with AN, and BN. A large proportion of the patients experienced clinically significant improvements in ED symptoms after the treatment. At last, the third study found that a group-based CFT combined with evidence-based outpatient treatment as usual for ED generated greater improvements in self-compassion, fears of self-compassion, fears of receiving compassion, shame, and ED pathology after treatment than treatment as usual alone (Kelly et al., 2017).

Summed up, these studies support the application of CFT to the ED. However, the studies are either brief, self-help in nature, examine individuals with BED only, have small samples, or examine CFT that is offered in conjunction with other treatments. As such, only tenuous conclusions can be drawn about the benefits of CFT for individuals with ED across diagnostic subtypes. Clearly, there is a need for a randomized controlled trial to test the efficacy of CFT for this population.

A general critique of psychological treatments is that “after decades of psychotherapy research and thousands of studies, there is no evidence based explanation for how or why even our most well-studied interventions produce change, that is, the mechanisms through which treatments operate” (Kazdin, 2009). This criticism is also valid for EDs, especially patients with EDs and childhood trauma. Therefore, it is a need for more research into the therapeutic qualities and interventions that are most associated with good outcomes, both at termination of therapy, and after a follow-up period. In particular, it would be helpful to know whether it is the quality of the therapeutic alliance or the specific process, e.g., self-compassion or cognitions that alleviate ED symptoms.

The proposed study is a randomized controlled trial, comparing outcome, and mechanisms of change in CFT and CBT for patients with ED, with and without childhood trauma. Outcome is examined in terms of ED symptom severity, trauma symptoms, general symptoms, and quality of life. The studied mechanisms are self-compassion, cognitions and alliance. Mechanism variables will be measured repeatedly (weekly) over the course of treatment.

### Objective

The first objective of this study is to assess the relative effects CBT and CFT in terms of recovery from ED defined as change from baseline to post-treatment in the global score of EDE. The mean scores in the two intervention groups are compared. The second objective is to assess whether – for patients with trauma compared to those without – CFT is more effective in (i) reducing ED psychopathology, (ii) reducing general symptoms and trauma symptoms, and (iii) improving health-related quality of life. The third objective is to examine the process of therapy by assessing self-compassion, cognitions, and alliance repeatedly over the course of treatment to examine their possible predictive and mediating effects on treatment outcome.

## Methods

### Design

**FIGURE 1:**
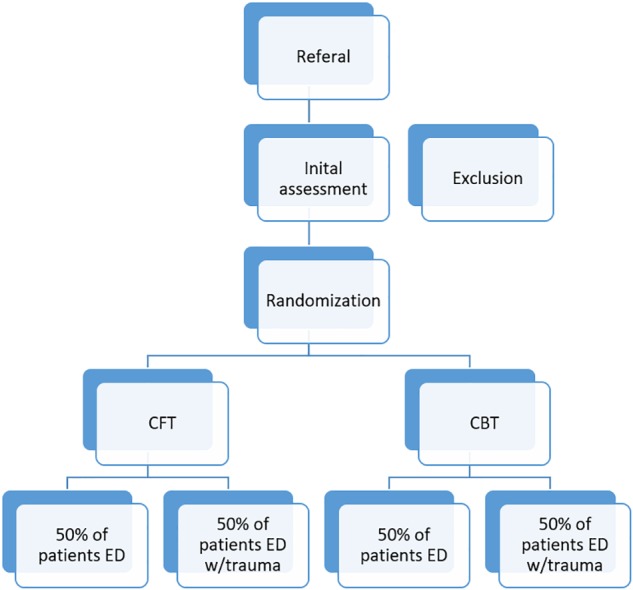
The figure illustrates the recruitment, screening, and randomization of participants to the project. Exclusion and inclusion criteria are reported in the text.

This research project involves a randomized controlled trial using a naturalistic sample of patients with ED, with and without childhood trauma. Patients will be randomly assigned to CFT or CBT. Every 3–4 months (from January 2015 – January 2019), 16 new patients will be admitted for 13 weeks of inpatient treatment in ED Unit at Modum Bad Hospital. Assessment on the overall outcome measures will take place at the pre-care evaluation, at admission, discharge, and 12-month follow-up. The weekly outcome and process measures will be completed every Monday. The present study protocol was written in accordance with the standard protocol items: recommendations for interventional trials (SPIRIT) (Chan et al., 2015) (see Figure 1 and Supplementary Material).

### Participants

Potential participants will initially be evaluated by a team of independent psychologists and psychiatrists who have been trained and have experience in diagnostic assessment. Consensus discussions with an experienced therapist will be used in the case of diagnostic ambiguity. Those who meet criteria for admission into the program will be put on a waiting list. The average waiting time between the pre-care evaluation and the admission evaluation is projected to be 10–15 weeks.

#### Inclusion Criteria

In order to be eligible to participate in this study, a participant must meet all of the following criteria: (1) adult inpatients (from age 18 years) with an ED diagnosis: AN, BN, and OSFED (EDNOS), according to the eating disorder examination-interview and (2) provision of informed consent.

#### Exclusion Criteria

A potential participant who meets any of the following criteria will be excluded from participation in this study: (1) current suicidal risk or psychosis that need extensive care, (2) serious substance abuse interfering with treatment, or (3) ongoing trauma (e.g., current involvement in an abusive relationship).

### Randomization

Patients will complete the baseline measures and after having signed an informed consent, a researcher blind to the study condition will conduct the random assignment procedure. A blocked randomization procedure will be used to ensure that there will be assigned an equal number of patients with trauma and non-trauma to each condition. Hence the blocks are in pair, such as two trauma patients and two non-trauma patients will be randomly assigned to each condition. The probability for each patient to end up in any of the two conditions will be kept constant at 0.5.

Researchers in the study will be blind to trauma category and the patient’s allocation to the groups. Because of the nature of the treatment content and procedures, therapists, and patients will not be blinded to the group allocation. Patients are informed about their group allocation after completing the pre-care evaluation.

### Statistical Methods

We will conduct independent *t*-test with the continuous variables and chi-square test with the categorical variables to check for differences at baseline. Treatment differences will be analyzed using hierarchical linear modeling (HLM; (Raudenbush and Bryk, 2002), hence all available data will be used. Thus, a patient with only data at admission can be included in an analysis and contribute to the estimation of model parameters (Kwok et al., 2008). Effect sizes (Cohen’s *d*) for HLM-analysis will be performed according to Feingold (2009). Interpretations will be based on the categorization of Cohen (1988).

Frequent longitudinal measurements in the treatment period will be analyzed disaggregating between patient effects (i.e., a patients standing relative to other patients) from the effect of individual patients development throughout therapy (i.e., a particular patients deviation from that patients expected level at a specific time point) using longitudinal mixed models (Curran and Bauer, 2011). This disaggregation is able to examine intra-individual process of change that is not contaminated with between-person variability (Paccagnella, 2006; Enders and Tofighi, 2007; Curran and Bauer, 2011). Within effects gives information on the level of the individual patient, making research findings directly applicable for therapists working with a specific patient.

Moderator variable will be included to analyze whether any treatment effects are modified by trauma or ED diagnosis. If treatment arms do not differ in outcome, combined latent class growth curve modeling will be conducted to analyze reasons for individual differences at baseline and in the rate of change across time. The multiple imputation procedure in SPSS will be used to impute missing data, if the data is missing at random.

### Power Estimation

A lower-bound probability of detecting effects in clinical research (power) of 0.80 has been suggested (Cohen, 1988). To get some idea of the statistical power in the present study, we estimated the necessary number of observations (*n*) for achieving a power of 0.80 of detecting a medium effect (Cohen’s *d* = 0.5) using an alpha level of 0.05 for three common tests: Calculations of statistical power were performed with G^∗^Power (Buchner and Erdfelder, 1996). All other statistical analyses were conducted with SPSS.

The primary outcomes in the two groups were compared using analysis of covariance (ANCOVA) with pre-treatment values as the covariate. Making the conventional assumptions of no treatment by pre-treatment interaction, this may be formulated as:

(1) $${\text{Post}}_{\text{i}}=\alpha +\beta_{1}^{*}{\text{Treatment}}_{\text{i}}+\beta_{2}^{*}{\text{Pre}}_{i}+\varepsilon_{\text{i}}$$

The treatment-effect may be estimated by the increase in explained variance when including the Treatment effect in the model. Using *d* = 0.5 this corresponds to an increase in *R*^2^ of 0.06. Assuming a rejection criterion (alpha) of 0.05 and a desired power of 0.80 of detecting an increase in *R*^2^ of 0.06, the estimated required sample size is 62 patients per treatment condition.

The design also included a dichotomous “trauma” condition (see section “Introduction”). Reasoning as above the model may be formulated as:

(2) $${\text{Post}}_{\text{i}}=\alpha +\beta_{1}^{*}{\text{Treatment}}_{\text{i}}+\beta_{2}^{*}{\text{Trauma}}_{\text{i}}+\beta_{3}^{*}{\text{(Treatment}}_{\text{i}}^{*}{\text{Trauma}}_{\text{i}}\text{)}+\beta_{4}^{*}{\text{Pre}}_{i}+\varepsilon_{\text{i}}$$

The effect of interest, the Treatment by Trauma interaction, will be estimated by the increase in explained variance when including this effect in the model over a model with all other effects included. Assuming a rejection criterion (alpha) of 0.05 and a desired power of 0.80 of detecting an increase in *R*^2^ of 0.06, the estimated required sample size is 64 patients per treatment condition. The statistical power of the present study with total *N* = 144 thus appears adequate.

As there seems to be no single standard for power analysis within disaggregating between patient effects from the effect of individual patients using longitudinal mixed models, we did not conduct further power, or sample size for this analysis.

### Treatments

#### Structure

The treatment in both conditions is a multicomponent inpatient program, components including a combination of small groups, individual, and milieu therapy lasting for 13 consecutive weeks, with two of these weeks the patients have a home stay where they practice new behavior. The treatment is closed, that is the patients start and end treatment at the same time and the group is not open to new patients if others end treatment. There are six group therapy sessions each week, altogether 66 group sessions. The duration of group session is 90 min. There are three individual therapy sessions each week, two of them with the individual therapist, and one with the milieu therapist, altogether 33 individual session. The duration of the individual session is 55 min. In these group and the individual sessions, the therapists deliver specific CBT or CFT treatment delivered in accordance with the respective treatment protocols, the manuals for which are available upon request from the first author. In addition to the specific therapy, both groups will participate in physical activity for 90 min and a community group meeting for 60 min each week.

#### Common Content

Both conditions have a focus on the working alliance in which therapist and the patient collaborate to overcome the ED. Underweight patients are encouraged to be active and responsible during the process of weight regain and weight maintenance. If these patients feel controlled it tends to increase their resistance. Both conditions are in addition designed to replace dysfunctional eating patterns with normal eating habits, and develop strategies for resisting binging and purging. The patients have to eat sufficient food to meet the body’s demands for energy. Ongoing self-monitoring and the accomplishment of planned homework assignment at the hospital are of importance to achieve and maintain the change.

#### Specific Content of Cognitive Behavioral Therapy (CBT)

The cognitive behavioral therapy treatment used in this trial is an adaptation of outpatient CBT for ED, postulated by Waller et al. (2007). It uses the same procedures and strategies but it is more intensive. It is primarily concerned with the processes that are maintaining the patients’ ED psychopathology, with cognitive processes being viewed of central importance. It uses cognitive, behavioral, and psychoeducational strategies. The treatment is suitable for all forms of ED diagnosis, as long as inpatient management is appropriate. The specific ED diagnosis is not of relevance to the treatment. It is the defined problems present and the processes that appear to be maintaining them that guide the content of the treatment. The key strategy of CBT is to create a case formulation of the maintaining mechanisms of the patients’ psychopathology. The formulation is used to identify the features to address in the treatment. An initial personal formulation is built with patients in the beginning of the treatment, but is revised during the treatment if necessary. If the patients have trauma symptoms that maintain their ED, and the patients do imaginal exposure and/or imagery rescripting. The treatment is designed to reduce ED symptoms and enhance their control over life but is also concerned with the entire psychosocial functioning of the patients (see Table 1).

**Table 1:** Differences in approach and interventions used in cognitive-behavioral therapy and compassion-focused therapy.

Approach used	CBT	CFT
Psychoeducational interventions	• Educate about the nature of CBT • Anxiety management training, relaxation, and distraction techniques	• Educate about the nature of CFT • Mindfulness – learning how to pay attention in the present moment • Understanding symptoms (e.g., restricting or binging) as related to safety and emotion regulation strategies • Understanding the origins and functions of self-criticism, shame, and pride • Learning to observe self with self-kindness and warmth
In-session work	• Socratic questions, focus toward overevaluation of eating, shape and weight, and schema level beliefs • Cognitive restructuring to examine core beliefs, dysfunctional assumptions, and negative automatic thoughts • Challenging maintaining behaviors and physical states • If trauma symptoms maintain ED; imaginal exposure of trauma scenes	• Developing compassionate focus using a variety of interventions, including compassionate imagery, thinking, behavior, or emotion • Working on the fears and blocks to developing compassion • Socratic questions, focus toward self-critical thoughts • Compassionate letter writing – focusing on being kind, supportive, and nurturing Develop sensitivity and acceptance into one’s own difficulties through self-reflection
Behavioral experiments	• Use of exposure therapy, meal planning • Exposure and making predictions toward meals, weight, and shape	• Exposure toward meals, weight, and shape with focus toward exploring what the compassionate self could say/do in the situation to help
Self-monitoring	• Completion of thought records, charts, cost benefit analysis to explore emotions thoughts, behavior, and bodily reactions	• Examining positives, e.g., what went well, and focusing on specific qualities • Use of self-compassion diary

**Table 2:** Method and timing of assessment.

Measure	Initial assessment	Start of treatment	During treatment	End of treatment	Follow-up
Sociodemographic data	×	×		×	×
MINI	×				×
EDI-I	×	×		×	×
CTQ	×				
EDE-Q	×	×	×	×	×
SCL-90R	×	×		×	×
EDI-2	×	×		×	×
IIP	×	×		×	×
BDI	×	×		×	×
SF-36	×	×		×	×
PSS-SR	×	×	×	×	×
WAI			×		
SCS	×	×	×	×	×
ATQ			×		

*EDE-I, eating disorder examination interview; MINI, mini international neuropsychiatric Interview; CTQ, childhood traumatic questionnaire; SCL-90R, symptom check list 90 revised; EDE-Q, eating disorder examination questionnaire; EDI, eating disorder inventory; IIP, inventory of interpersonal problems; BDI, beck depression inventory; SF-36, short form health survey; PSS-SR, post traumatic symptom scale self-rating; WAI, working alliance inventory; SCS, self-compassion scale; SCL-5; symptom checklist-5; ATQ, automatic thought questionnaire; HRV; heart rate variability.*

#### Specific Content of Compassion-Focused Therapy (CFT)

The compassion-focused therapy treatment used in this trial is an adaptation of outpatient CFT for ED, postulated by Gale et al. (2014) and Goss and Allan (2014). It uses the same procedures and strategies but it is more intensive. CFT offers a structured approach to help patients gain control of their chaotic eating patterns, trauma symptoms (for trauma patients), and the processes that underlie them. It is primarily concerned with the processes of developing a compassionate approach to managing the physical and emotional demands of following a structured eating program and to reduce shame, in particular body shame.

To increase compassion and reduce shame, compassionate mind training (CMT) is a central part of the program. CMT has two main aims, the first is to help patients develop their soothing system, and use this to regulate other motivational systems and affective states (e.g., feat, anger, or disgust). The second is to use a range of techniques and group work to help patients develop a compassionate motivational system and develop their capacities of giving compassion to others, receiving compassion from others, and for self-compassion. It is especially focused on helping the patients imagine a future in which they can be motivated by compassion and no longer need their ED. It also helps to identify and work with blocks to feeling safe and experiencing compassion from others and compassion for the self. Because their “compassion” systems are so under-used and unfamiliar they often find this very challenging, particularly if their self-identity, and sense of pride has been linked to their ED. CMT uses imagery tasks that are designed to give space to experience and practice generating specific types of motivations and emotions associated with compassion.

One of the key foci of these exercises is to help the patient develop a more compassionate relationship with themselves. Within the treatment program, this is specifically targeted at managing ED symptoms, the issues that trigger them, and the functions they serve. Thus, we would explore questions such as “How would a compassionate person help you to eat?” or “What compassionate things could you do or say to help you eat breakfast?” The key is to develop coping thoughts and response that are “felt” to be helpful, to enable patients to let go of ED behaviors that have come to feel “safe” ways of managing difficult emotions or experiences and to develop more “self-caring” behaviors in everyday life (see Table 1).

### Screening Instrument and Primary Treatment Outcome

The trial uses standardized instruments with good psychometric properties and high clinical utility. Demographic variables (age, sex, social conditions, education, and connection to the labor market), previous treatment, medication, BMI, and pregnancy are assessed at the first assessment interview with a psychologist and by a psychiatrist or attending physician at their assessment interview (part of the clinical routine) (see Table 2).

#### Mini International Neuropsychiatric Interview (MINI) (Sheehan et al., 1998)

Mini international neuropsychiatric interview is used to assess present psychosis, depression, suicidal risk and abuse of alcohol, medicine and/or narcotics. MINI is a short structured diagnostic interview compatible with international diagnostic criteria, including the international classification of diseases (ICD-10) and the Diagnostic and Statistical Manual of Mental Disorders (DSM-IV).

#### Eating Disorder Examination – Interview (EDE) (Fairburn et al., 2008)

Eating disorder examination will be used in order both to obtain ED diagnosis for inclusion and as a treatment outcome measuring at discharge and 12-months follow-up. We use the authorized Norwegian version (version 12 and 17). These interviews are performed by four interviewers trained by an experienced clinician and researcher. The assessors are blind to the patients’ treatment condition and have no involvement with treatment. Two primary outcome variables will be generated from the EDE ratings: change in the severity of ED features as measured by the global EDE score, and having a global EDE score less than one standard deviation above the community mean (i.e., below 1.74) (Kendall et al., 1999). Normative comparisons of this type are widely used to identify clinically significant change (Kendall et al., 1999; Ogles et al., 2001; Kazdin, 2003).

#### Childhood Trauma Questionnaire (CTQ) (Bernstein et al., 2003)

Childhood trauma questionnaire is used to obtain measures of childhood trauma. This is a questionnaire inquiries about childhood maltreatment in five areas, hence severity of sexual, physical, and emotional abuse, as well as physical and emotional neglect. Each subscale has five items graded on a 5-point Likert scale (1 = never true, 5 = very often true). CTQ holds satisfactory psychometric properties across Norwegian samples (Dovran et al., 2013). To identify possible cases of trauma, we will also use this instrument to define accurate case determination with the recommended scoring options by Walker et al. (1999). Based on a receiver operating characteristic methods they determined threshold scores for each of the five subscales that provided very good to excellent sensitivity and specificity (≥0.85) for each of the five subscales. Patients who score ≥8 on the sexual abuse, physical abuse or physical neglect scale or ≥10 at emotional abuse scale or ≥15 at emotional neglect scale will be placed in the trauma group. The remaining patients, with scores below the thresholds for all five maltreatment groups, will be considered to be a non-trauma group. This last group contains patients with a range of maltreatment experiences from none to just below. In case of ambiguity regarding case determination, it will be resolved by consensus discussions with an experienced therapist independent of the study.

### Secondary Outcome Measurements

#### The Eating Disorder Examination-Questionnaire-6 (EDE-Q-6) (Fairburn et al., 1993)

Eating disorder examination-questionnaire is a self-report measure that was adapted from the interview-based EDE and measures ED pathology. The total score is used as an indicator for the level of ED pathology, with a higher score denoting more pathology.

#### Symptom Checklist-90-Revised (SCL-90) (Derogatis, 1983)

Symptom checklist-90-revised is a multidimensional patient-reported questionnaire for measuring psychological distress or the degree of affective distress. The global severity index (GSI), which is the global score covering all 90 items, will be used in the trial.

#### Eating Disorder Inventory-2 (EDI-2) (Garner et al., 1983)

Eating disorder inventory-2 consists of 64 questions related specifically to eating behaviors, body perception as well as more general questions about attitudes, behaviors, and emotions. A total sum score and eight subscores are calculated (Nevonen and Broberg, 2001).

#### Inventory of Interpersonal Problems (IIP-32) (Horowitz et al., 1988)

Inventory of interpersonal problems consists of 64 questions with eight subscales assessing interpersonal problems.

#### The Beck Depression Inventory II (BDI) (Beck et al., 1996)

Beck depression inventory II is a 21-item self-report scale measuring degree of depressive symptoms.

#### Short Form Health Survey (SF-36) (Ware and Sherbourne, 1992)

Short form health survey assesses health-related quality of life and health status. It is a multidimensional instrument, with 36 questions to measure eight dimensions.

#### PTSD Symptom Scale-Self-Rating (PSS-SR) (Foa et al., 1993)

PTSD symptom scale-self-rating is a self-report outcome measure and contains 17 items assessing the severity of the PTSD symptoms described in the DSM-IV.

### Process Measurements/Mediators

#### Working Alliance Inventory-12 (WAI) (Hatcher and Gillaspy, 2006)

Working alliance inventory-12 assesses the bond between patient and therapist, and their agreement on tasks and goals of therapy.

#### Self-Compassion Scale (SCS) (Neff, 2003)

Self-compassion scale assesses the degree of self-compassion. Subscale scores for self-kindness, selfjudgment, common humanity, isolation, mindfulness, and over-identification. Self-compassion are computed by averaging across items within each subscale.

#### The Automatic Thoughts Questionnaire (ATQ) (Hollon and Kendall, 1980)

Automatic thoughts questionnaire is one of the most widely used instruments for measuring automatic thought. We used the 8-item version of the ATQ in this study because the short version showed internal consistency and validity equivalent to the original ATQ-30 (Netemeyer et al., 2002).

### Treatment Integrity

Both the therapists in the CFT treatment and the CBT treatment have received supervision from the architect behind the CFT condition (K. Goss) and the architect behind the CBT condition (G. Waller), respectively, since spring 2015 and will continue to receive biweekly supervision throughout the research period. All therapists will thus have the same training in either CBT or CFT and fidelity with model will be ensured by regular expert supervision. Individual therapy sessions will be videotaped and rated for treatment competence and adherence to the treatment protocol.

## Discussion

This paper has provided a description of a trial comparing two treatment models, its rationale and methods. The aims of the study, is to evaluate the effect of two treatment models, hence CFT and CBT on ED with or without childhood trauma and to study therapy processes important for treatment outcome for these patients. This is the first randomized trial of the effect of CFT for a transdiagnostic sample of ED. A large sample will be recruited among patients from outpatient clinics. Few exclusion criteria are applied and in this sense, the sample is clinically relevant and hence will enhance the external validity.

Each approach may yield efficacy for patients with ED without trauma. In that sense, it will provide support for CFT as an evidence supported treatments for EDs in addition to CBT. This is an important accomplishment given the complexity of these disorders. An important additional task is to examine if CFT could be more effective in reducing ED psychopathology, general symptoms and trauma symptoms and improving health-related quality of life for patients with additional childhood trauma experiences. By put forward knowledge of who responds best to which kind of treatment, we may offer help to a larger proportion of sufferers of ED.

In the present trial it is possible to explore the mediation effects of mechanisms. This will add to our knowledge about the relative effects of treatments for ED, compassion, cognitions, and therapeutic alliance. For EDs the influence of such mechanisms of change in CBT treatment is less established (Wilson et al., 2007; Horvath et al., 2011; Brauhardt et al., 2014; Wampold, 2015; Zaitsoff et al., 2015), and in CFT it is less studied due to the fact that the treatment is relatively new. However, there are a few of CFT mechanism studies (Kelly et al., 2013, 2014a,b), however, these are mostly correlational studies and therefore limited in drawing conclusions. Important research step is to observe session by session both the specific (self-compassion and cognitions) and generic (alliance) processes and ED symptoms throughout the whole treatment period.

Strengths of this RCT include the multilevel outcome measures, which comprise several psychological variables as well as follow-up up to 1 year after treatment. In addition, the protocol includes measures of treatment adherence and fidelity, thus enabling precise replications and weekly measurements which allows for an examination of temporal sequence. This allows some causal inferences. There are, however, limitation to consider given the chosen research design. The present study has no control condition, such as a no treatment, waiting-list or placebo. This could be a limitation especially if we do not detect a difference in outcome between the two treatment conditions. If no difference in outcome will be found, it will be difficult to decide to what extent the effect of treatment has to be ascribed to non-specific factors, the effect of testing, or time. However, we could argue that the level of spontaneous recovery in severe, enduring, treatment resistant and comorbid ED patients is low (Touyz and Hay, 2015; Treasure et al., 2015). In addition, comparing established treatments with no treatment or waitlist control group could be considered unethical for such a severe patient group (Devilly and McFarlane, 2009).

In addition, the therapist that provide the treatment in the two conditions belong to the same unit. Both the patients and the therapists meet each other in common meetings and meals at the unit and this can be the source of contamination between the groups. However, neither the patients nor the therapists in one group are informed of the patients’ therapeutic process in the other groups and we therefore hypothesize that any contamination will be minor. Furthermore, the exposure toward meals, weight, and shape and Socratic questioning in CFT overlaps substantially with the methods and approach in the CBT. This can possibly confound the comparison, even though we have try to counteract for that by substantially supervision.

## Data Availability

The datasets that will be generated and/or analyzed during the current study will not be publicly available due to Norwegian laws and regulations, but are available from the corresponding author on reasonable request.

## Ethics Statement

This study was carried out in accordance with the recommendations of the South-Eastern Regional Committee for Medical and Health Research Ethics of Norway (SE-RCE, 2014/836-1) and is enrolled in the international database of controlled trials www.clinicaltrials.gov. The protocol was approved by the SE-RCE. All data will be de-identified by using a unique ID number linked to names. The list of ID numbers and names are only available for the PI, and its destruction upon final data inclusion completely anonymizes all data. All patients will be given written and orally information about the study and must sign an informed consent form in accordance with the Declaration of Helsinki before inclusion in the study. The information will address confidentiality and the patients possibility to withdraw their participation at any time and without clarification. Withdrawing their participation will not affect the treatment. The patients can also refuse to take part in the study, and still receive treatment from Modum Bad. No harm is expected from the treatment. In case of deterioration, the responsible therapist can advise to stop research participation at any time. The patients can address concerns and queries about the study throughout the whole research period to the project leader (KV).

## Author Contributions

KV had responsibility for the design and conduct of the study, co-developed the training, and revised the manuscript. AH and BW had responsibility for the design and implementation of the study and drafting and revising the manuscript. AH, BW, and DQ had major responsibility for drafting and revising the manuscript. KG and GW had substantial responsibility for the training and supervision of the therapists and drafting and revising the manuscript. All authors approved the final version of the manuscript and agreed to be accountable for the content of the work.

## Conflict of Interest Statement

The authors declare that the research was conducted in the absence of any commercial or financial relationships that could be construed as a potential conflict of interest.

## Abbreviations

AN: anorexia nervosa
BDI-II: beck depression inventory nb 2
BED: binge eating disorder
BMI: body mass index
BN: bulimia nervosa
CBT: cognitive-behavioral therapy
CFT: compassion-focused therapy
CIA: clinical impairment assessment
DSM-5: diagnostic and statistical manual nb. 5
ED: eating disorder
EDE-q: eating disorder examination questionnaire
EDI-2: eating disorder inventory-2
IIP-48: inventory of interpersonal problems
PSS-SR: post traumatic symptom scale self-report
SCL-90: symptom checklist-90
SCS: self compassion scale
WAI: working alliance inventory

## References

[B1] American Psychiatric Association [APA] (2006). Treatment of patients with eating disorders, American Psychiatric Association. *Am. J. Psyc.* 163 (Suppl. 7), 4. 16925191

[B2] American Psychiatric Association [APA] (2013). *Diagnostic and Statistical Manual of Mental Disorders (DSM-5^®^).* Philadelphia: American Psychiatric Pub.

[B3] Attia E. Becker A. E. Bryant-Waugh R. Hoek H. W. Kreipe R. E. Marcus M. D. (2013). Feeding and eating disorders in DSM-5. *Am. J. Psychiatry* 170 1237–1239. 10.1176/appi.ajp.2013.13030326 24185238

[B4] Beck A. T. Steer R. A. Brown G. K. (1996). Manual for the beck depression inventory-II. San Antonio, TX. *Psychol. Corpor.* 1:82.

[B5] Bernstein D. P. Stein J. A. Newcomb M. D. Walker E. Pogge D. Ahluvalia T. (2003). Development and validation of a brief screening version of the childhood trauma questionnaire. *Child Abuse Negl.* 27 169–190. 10.1016/s0145-2134(02)00541-0 12615092

[B6] Brauhardt A. de Zwaan M. Hilbert A. (2014). The therapeutic process in psychological treatments for eating disorders: a systematic review. *Int. J. Eat. Disord.* 47 565–584. 10.1002/eat.22287 24796817

[B7] Buchner A. Erdfelder E. (1996). On assumptions of, relations between, and evaluations of some process dissociation measurement models. *Conscious Cogn.* 5 581–594. 10.1006/ccog.1996.0034 9063617

[B8] Chan A. W. Tetzlaff J. M. Altman D. G. Laupacis A. Gotzsche P. C. Krle A. J. K. (2015). SPIRIT 2013 Statement: defining standard protocol items for clinical trials. *Rev. Panam Salud Publica* 38 506–514. 27440100 PMC5114122

[B9] Cohen J. (1988). *Statistical Power Analysis for the Behavioral Sciences*, 2nd Edn. Hillsdale, NJ: Lawrence Erlbaum Associates.

[B10] Curran P. J. Bauer D. J. (2011). The disaggregation of within-person and between-person effects in longitudinal models of change. *Annu. Rev. Psychol.* 62 583–619. 10.1146/annurev.psych.093008.100356 19575624 PMC3059070

[B11] Derogatis L. (1983). *Symptom Checklist 90-revised: Administration, Scoring and Procedures Manual II.* Towson. MD: Clinical Psychometry Research Center

[B12] Devilly G. J. McFarlane A. C. (2009). When wait lists are not feasible, nothing is a thing that does not need to be done. *J. Consult. Clin. Psychol.* 77 1159–1168. 10.1037/a0016878 19968391

[B13] Dovran A. Winje D. Overland S. N. Breivik K. Arefjord K. Dalsbo A. S. (2013). Psychometric properties of the Norwegian version of the Childhood Trauma Questionnaire in high-risk groups. *Scand. J. Psychol.* 54 286–291. 10.1111/sjop.12052 23672336

[B14] Enders C. K. Tofighi D. (2007). Centering predictor variables in cross-sectional multilevel models: a new look at an old issue. *Psychol. Methods* 12 121–138. 10.1037/1082-989X.12.2.121 17563168

[B15] Fairburn C. G. Cooper Z. O’connor M. E. (2008). “Eating disorder examination (Edition 16.0D),” in *Cognitive Behavior Therapy and Eatingdisorders*, ed. Fairburn C. G. (New York, NY: Guilford Press).

[B16] Fairburn C. G. Bohn K. (2005). Eating disorder NOS (EDNOS): an example of the troublesome “not otherwise specified” (NOS) category in DSM-IV. *Behav. Res. Ther.* 43 691–701. 10.1016/j.brat.2004.06.011 15890163 PMC2785872

[B17] Fairburn I. C. Wilson G. Schleimer K. (1993). *Binge Eating: Nature, Assessment and Treatment.* New York, NY: Guilford Press, 317–360.

[B18] Feingold A. (2009). Effect sizes for growth-modeling analysis for controlled clinical trials in the same metric as for classical analysis. *Psychol. Methods* 14 43–53. 10.1037/a0014699 19271847 PMC2712654

[B19] Foa E. B. Riggs D. S. Dancu C. V. Rothbaum B. O. (1993). Reliability and validity of a brief instrument for assessing post-traumatic stress disorder. *J. Traum. Stress* 6 459–473. 10.1007/bf00974317

[B20] Gale C. Gilbert P. Read N. Goss K. (2014). An evaluation of the impact of introducing compassion focused therapy to a standard treatment programme for people with eating disorders. *Clin. Psychol. Psychother.* 21 1–12. 10.1002/cpp.1806 22740105

[B21] Garner D. M. Olmstead M. P. Polivy J. (1983). Development and validation of a multidimensional eating disorder inventory for anorexia nervosa and bulimia. *Int. J. Eat Dis.* 2 15–34. 10.1002/1098-108x(198321)2:2<15::aid-eat2260020203>3.0.co;2-6

[B22] Gilbert P. (1998). “What is shame?,” in *Shame Interpersonal Behaviour, Psychopathology and Culture*, ed. Gilbert P. A. (New York, NY: Oxford University Press), 3–38.

[B23] Gilbert P. (2005). *Compassion: Conceptualisation, Research, and use in Psychotherapy.* London: Routledge.

[B24] Gilbert P. (2010). *The Compassionate Mind: A New Approach to Life’s Challenges.* London: New Harbinger Publications.

[B25] Gilbert P. D. (2007). Spirituality and mental health: a very preliminary overview. *Curr. Opin. Psych.* 20 594–598. 10.1097/yco.0b013e3282f0eee1 17921761

[B26] Goss K. Allan S. (2009). Shame, pride and eating disorders. *Clin. Psychol. Psychother.* 16 303–316. 10.1002/cpp.627 19639646

[B27] Goss K. Allan S. (2014). The development and application of compassion-focused therapy for eating disorders (CFT-E). *Br. J. Clin. Psychol.* 53 62–77. 10.1111/bjc.12039 24588762

[B28] Goss K. Gilbert P. (2002). “Eating disorders, shame and pride: a cognitive-behavioural functional analysis,” in *Body Shame: Conceptualisation, Research and Treatment*, eds Gilbert P. Miles J. (New York, NY: Brunner-Routledge), 219–255.

[B29] Hatcher R. L. Gillaspy J. A. (2006). Development and validation of a revised short version of the working alliance inventory. *Psychother. Res.* 16 12–25. 10.1080/10503300500352500

[B30] Hay P. (2013). A systematic review of evidence for psychological treatments in eating disorders: 2005-2012. *Int. J. Eat. Disord.* 46 462–469. 10.1002/eat.22103 23658093

[B31] Hoffart A. Oktedalen T. Langkaas T. F. (2015). Self-compassion influences PTSD symptoms in the process of change in trauma-focused cognitive-behavioral therapies: a study of within-person processes. *Front. Psychol.* 6:1273. 10.3389/fpsyg.2015.01273 26379596 PMC4551056

[B32] Hollon S. D. Kendall P. C. (1980). Cognitive self-statements in depression: development of an automatic thoughts questionnaire. *Cog. Ther. Res.* 4 383–395. 10.1007/bf01178214 9420353

[B33] Horowitz L. M. Rosenberg S. E. Baer B. A. Ureno G. Villasenor V. S. (1988). Inventory of interpersonal problems: psychometric properties and clinical applications. *J. Consult. Clin. Psychol.* 56 885–892. 10.1037//0022-006x.56.6.885 3204198

[B34] Horvath A. O. Del Re A. C. Fluckiger C. Symonds D. (2011). Alliance in individual psychotherapy. *Psychotherapy* 48 9–16. 10.1037/a0022186 21401269

[B35] Hudson J. I. Hiripi E. Pope H. G. Jr. Kessler R. C. (2007). The prevalence and correlates of eating disorders in the National Comorbidity Survey Replication. *Biol. Psychiatry* 61 348–358. 10.1016/j.biopsych.2006.03.040 16815322 PMC1892232

[B36] Irons C. Gilbert P. (2005). Evolved mechanisms in adolescent anxiety and depression symptoms: the role of the attachment and social rank systems. *J. Adolesc.* 28 325–341. 10.1016/j.adolescence.2004.07.004 15925685

[B37] Jenkins P. E. Hoste R. R. Meyer C. Blissett J. M. (2011). Eating disorders and quality of life: a review of the literature. *Clin. Psychol. Rev.* 31 113–121. 10.1016/j.cpr.2010.08.003 20817335

[B38] Kazdin A. E. (2003). *Research Design in Clinical Psychology*, 4th Edn. Boston: Allyn and Bacon.

[B39] Kazdin A. E. (2009). Understanding how and why psychotherapy leads to change. *Psychother. Res.* 19 418–428. 10.1080/10503300802448899 19034715

[B40] Kelly A. Carter J. Zuroff D. Borairi S. (2013). Self-compassion and fear of self-compassion interact to predict response to eating disorders treatment: A preliminary investigation. *Psychother. Res.* 23 252–264. 10.1080/10503307.2012.717310 22917037

[B41] Kelly A. C. Carter J. C. (2015). Self-compassion training for binge eating disorder: a pilot randomized controlled trial. *Psychol. Psychother.* 88 285–303. 10.1111/papt.12044 25330466

[B42] Kelly A. C. Carter J. C. Borairi S. (2014a). Are improvements in shame and self-compassion early in eating disorders treatment associated with better patient outcomes? *Int. J. Eat Disord.* 47 54–64. 10.1002/eat.22196 24115289

[B43] Kelly A. C. Vimalakanthan K. Carter J. C. (2014b). Understanding the roles of self-esteem, self-compassion, and fear of self-compassion in eating disorder pathology: an examination of female students and eating disorder patients. *Eat Behav.* 15 388–391. 10.1016/j.eatbeh.2014.04.008 25064287

[B44] Kelly A. C. Wisniewski L. Martin-Wagar C. Hoffman E. (2017). Group-based compassion-focused therapy as an adjunct to outpatient treatment for eating disorders: a pilot randomized controlled trial. *Clin. Psychol. Psychother.* 24 475–487. 10.1002/cpp.2018 27237928

[B45] Kendall P. C. Marrs-Garcia A. Nath S. R. Sheldrick R. C. (1999). Normative comparisons for the evaluation of clinical significance. *J. Consult. Clin. Psychol.* 67 285–299. 10.1037//0022-006x.67.3.285 10369049

[B46] Kwok O. M. Underhill A. T. Berry J. W. Luo W. Elliott T. R. Yoon M. (2008). Analyzing longitudinal data with multilevel models: an example with individuals living with lower extremity intra-articular fractures. *Rehabil. Psychol.* 53 370–386. 10.1037/a0012765 19649151 PMC2613314

[B47] Lee D. A. Scragg P. Turner S. (2001). The role of shame and guilt in traumatic events: a clinical model of shame-based and guilt-based PTSD. *Br. J. Med. Psychol.* 74(Pt 4), 451–466. 10.1348/000711201161109 11780793

[B48] Lutz A. Greischar L. L. Rawlings N. B. Ricard M. Davidson R. J. (2004). Long-term meditators self-induce high-amplitude gamma synchrony during mental practice. *Proc. Natl. Acad. Sci. U.S.A.* 101 16369–16373. 10.1073/pnas.0407401101 15534199 PMC526201

[B49] Machado P. P. Machado B. C. Goncalves S. Hoek H. W. (2007). The prevalence of eating disorders not otherwise specified. *Int. J. Eat Disord.* 40 212–217. 10.1002/eat.20358 17173324

[B50] Mahon J. Bradley S. N. Harvey P. K. Winston A. P. Palmer R. L. (2001). Childhood trauma has dose-effect relationship with dropping out from psychotherapeutic treatment for bulimia nervosa: a replication. *Int. J. Eat. Disord.* 30 138–148. 10.1002/eat.1066 11449447

[B51] Mitchison D. Hay P. Slewa-Younan S. Mond J. (2012). Time trends in population prevalence of eating disorder behaviors and their relationship to quality of life. *PLoS One* 7:e48450. 10.1371/journal.pone.0048450 23144886 PMC3492397

[B52] National Collaborating Centre for Mental Health (2004). *Eating Disorders: Core Interventions in the Treatment and Management of Anorexia Nervosa, Bulimia Nervosa and Related Eating Disorders.* Leicester: British Psychological Society. 23346610

[B53] Neff K. (2003). The Development and validation of a scale to measure self-compassion. *Self Ident.* 2 223–250. 10.1080/15298860309027 26979311

[B54] Netemeyer R. G. Williamson D. A. Burton S. Biswas D. Jindal S. Landreth S. (2002). Psychometric properties of shortened versions of the automatic thoughts questionnaire. *Educ. Psychol. Meas* 62 111–129. 10.1177/0013164402062001008

[B55] Nevonen L. Broberg A. G. (2001). Validating the Eating Disorder Inventory-2 (EDI-2) in Sweden. *Eat. Weight. Disord.* 6 59–67. 10.1007/bf03339754 11456423

[B56] Ogles B. M. Lunnen K. M. Bonesteel K. (2001). Clinical significance: history, application, and current practice. *Clin. Psychol. Rev.* 21 421–446. 11288608 10.1016/s0272-7358(99)00058-6

[B57] Paccagnella O. (2006). Centering or not centering in multilevel models? The role of the group mean and the assessment of group effects. *Eval. Rev.* 30 66–85. 10.1177/0193841X05275649 16394187

[B58] Raudenbush S. W. Bryk A. S. (2002). *Hierarchical Linear Models: Applications and Data Analysis Methods.* Newcastle upon Tyne: Sage Publications.

[B59] Rodriguez M. Perez V. Garcia Y. (2005). Impact of traumatic experiences and violent acts upon response to treatment of a sample of Colombian women with eating disorders. *Int. J. Eat. Disord.* 37 299–306. 10.1002/eat.20091 15856503

[B60] Sheehan D. V. Lecrubier Y. Sheehan K. H. Amorim P. Janavs J. Weiller E. (1998). The Mini-International Neuropsychiatric Interview (M.I.N.I.): the development and validation of a structured diagnostic psychiatric interview for DSM-IV and ICD-10. *J. Clin. Psychiatry* 59 (Suppl. 20), 22–33. 9881538

[B61] Spielmans G. I. Benish S. G. Marin C. Bowman W. M. Menster M. Wheeler A. J. (2013). Specificity of psychological treatments for bulimia nervosa and binge eating disorder? a meta-analysis of direct comparisons. *Clin. Psychol. Rev.* 33 460–469. 10.1016/j.cpr.2013.01.008 23454220

[B62] Stice E. Marti C. N. Rohde P. (2013). Prevalence, incidence, impairment, and course of the proposed DSM-5 eating disorder diagnoses in an 8-year prospective community study of young women. *J. Abnorm. Psychol.* 122 445–457. 10.1037/a0030679 23148784 PMC3980846

[B63] Touyz S. Hay P. (2015). Severe and enduring anorexia nervosa (SE-AN): in search of a new paradigm. *J. Eat Disord.* 3 1–3. 26236477 10.1186/s40337-015-0065-z PMC4521346

[B64] Treasure J. Stein D. Maguire S. (2015). Has the time come for a staging model to map the course of eating disorders from high risk to severe enduring illness? an examination of the evidence. *Early Interv. Psych.* 9 173–184. 10.1111/eip.12170 25263388

[B65] Vrabel K. R. Hoffart A. Ro O. Martinsen E. W. Rosenvinge J. H. (2010). Co-occurrence of avoidant personality disorder and child sexual abuse predicts poor outcome in long-standing eating disorder. *J. Abnorm. Psychol.* 119 623–629. 10.1037/a0019857 20677852

[B66] Walker E. A. Gelfand A. Katon W. J. Koss M. P. Von Korff M. Bernstein D. (1999). Adult health status of women with histories of childhood abuse and neglect. *Am. J. Med.* 107 332–339. 10.1016/s0002-9343(99)00235-1 10527034

[B67] Waller G. Cordery H. Corstorphine E. Hinrichsen H. Lawson R. Mountford V. (2007). *Cognitive Behavioral Therapy for Eating Disorders A Comprehensive Treatment Guide.* Cambridge: Cambridge University Press.

[B68] Wampold B. E. (2015). How important are the common factors in psychotherapy? An update. *World Psychiatry* 14 270–277. 10.1002/wps.20238 26407772 PMC4592639

[B69] Ware J. E. Jr. Sherbourne C. D. (1992). The MOS 36-item short-form health survey (SF-36). I. conceptual framework and item selection. *Med. Care* 30 473–483. 10.1097/00005650-199206000-00002 1593914

[B70] Wilson G. T. Grilo C. M. Vitousek K. M. (2007). Psychological treatment of eating disorders. *Am. Psychol.* 62 199–216. 10.1037/0003-066X.62.3.199 17469898

[B71] Zaitsoff S. Pullmer R. Cyr M. Aime H. (2015). The role of the therapeutic alliance in eating disorder treatment outcomes: a systematic review. *Eat Disord.* 23 99–114. 10.1080/10640266.2014.9646236 25330409

